# Significant and Distinctive *n*-Grams in Oncology Notes: A Text-Mining Method to Analyze the Effect of OpenNotes on Clinical Documentation

**DOI:** 10.1200/CCI.19.00012

**Published:** 2019-06-11

**Authors:** Maryam Rahimian, Jeremy L. Warner, Sandeep K. Jain, Roger B. Davis, Jessica A. Zerillo, Robin M. Joyce

**Affiliations:** ^1^ Beth Israel Deaconess Medical Center and Harvard Medical School, Boston, MA; ^2^ Vanderbilt University Medical Center, Nashville, TN; ^3^ Vanderbilt University, Nashville, TN; ^4^ St Louis University, St Louis, MO

## Abstract

**PURPOSE:**

OpenNotes is a national movement established in 2010 that gives patients access to their visit notes through online patient portals, and its goal is to improve transparency and communication. To determine whether granting patients access to their medical notes will have a measurable effect on provider behavior, we developed novel methods to quantify changes in the length and frequency of use of *n*-grams (sets of words used in exact sequence) in the notes.

**METHODS:**

We analyzed 102,135 notes of 36 hematology/oncology clinicians before and after the OpenNotes debut at Beth Israel Deaconess Medical Center. We applied methods to quantify changes in the length and frequency of use of sequential co-occurrence of words (*n*-grams) in the unstructured content of the notes by unsupervised hierarchical clustering and proportional analysis of *n*-grams.

**RESULTS:**

The number of significant *n*-grams averaged over all providers did not change, but for individual providers, there were significant changes. That is, all significant observed changes were provider specific. We identified eight providers who were late note signers. This group significantly reduced its late signing behavior after OpenNotes implementation.

**CONCLUSION:**

Although the number of significant *n*-grams averaged over all providers did not change, our text-mining method detected major content changes in specific providers’ documentation at the *n*-gram level. The method successfully identified a group of providers who decreased their late note signing behavior.

## INTRODUCTION

History has documented numerous efforts toward enabling patients to become more engaged in their own care. After development of the clinical record in the 19th century in America,^1-7^ the 1973 Patient’s Bill of Rights^8^ is considered one of the first major steps to give patients the right to receive considerate and high-quality care and to access the details and written records about that care.^8^ Just over 20 years later, the 1996 Health Insurance Portability and Accountability Act established national standards for protecting the privacy of patient health data.^9,10^ Coincident with the enactment of the Health Insurance Portability and Accountability Act was the rise in personal home computing and Internet access, which allowed for the development of online patient portals where patients could access summary information about their medications, immunizations, visits, and laboratory results online.^11,12^ However, barriers still existed to patients viewing important parts of their health record, such as clinic visit notes and correspondence with consultants.

In 2010, OpenNotes was launched as a national initiative to promote greater transparency in doctor-patient communication. Patients received open access to unstructured clinician notes in their electronic health records through online patient portals. OpenNotes began as a 12-month demonstration project with primary care physicians at three US institutions.^13^ In surveys at the end of the pilot period, participating patients and doctors reported favorably on their experiences.^14^ Since the original study, more than 100 institutions with over 30 million patients have implemented OpenNotes.^15^

Despite positive initial findings, some doctors expressed concerns about unintended consequences. One ongoing concern about OpenNotes is whether the phenomenon of patients having access to this information would change how doctors constructed notes. One field of medical practice that is likely to have been affected by OpenNotes is hematology/oncology. We hypothesized that granting patients with cancer full access to their health records would influence providers to alter their documentation of patient encounters, given the sensitive and potentially anxiety-provoking nature of cancer diagnosis, the team-based nature of cancer care, and the importance of such notes for documenting communication between clinicians as well as between clinician and patient.

Most OpenNotes research to date has relied on surveys and subjective assessments. In previous preliminary work, we conducted to our knowledge the first objective assessment using visual analytic techniques to show that certain single-word co-occurrences had statistically significant changes before and after OpenNotes.^16^ In the current article, we describe methods to quantify changes in the length and frequency of sequential co-occurrence of words (*n*-grams) in the unstructured content of clinical notes by unsupervised hierarchical clustering and proportional analysis. We sought to explore quantitatively whether the introduction of OpenNotes has changed documentation of patient encounters on the basis of repeating sequential occurrences of words (*n*-grams) and modes of expression as seen in cluster/clique formation. Most providers use a combination of specific words, templates, and their own unique expressions when completing patient notes, and patterns in the unstructured content of these providers’ notes can be used to track documentation changes over time. We hypothesized that changes in the frequency of common phrases may act as a bellwether for changes in institutional attitudes and/or policy, reflect changing social norms and customs among providers, and/or reflect changes in administrative procedures.

## METHODS

Numerical analyses were performed using R version 3.3.1 and RStudio version 0.99.903 software (https://cran.r-project.org/bin/macosx/). The R libraries data.table, stringr, and edgebundleR were used. This study was approved by the Beth Israel Deaconess Medical Center (BIDMC) institutional review board (#2014P000158).

### Data Sources and Inclusion Criteria

Notes written by providers in the hematology/oncology department from January 1, 2012, to September 1, 2016, were retrieved. These dates bracket the November 25, 2013, OpenNotes rollout date for hematology/oncology clinics. The post-rollout period is longer than the antecedent period to capture the dynamics and transients of adoption. The antecedent period of 18 or more months was believed to be long enough to sample the stationary behavior of the unperturbed system. Analysis was restricted to initial notes, progress notes, and letters written by full-time medical doctors/doctors of osteopathic medicine and nurse practitioners; part-time faculty, fellows, and trainees were excluded because their documentation style might have changed over time as a result of on-the-job training. Finally, analysis was restricted to providers who wrote at least 100 (progress/initial) notes before and 100 notes after the rollout date. The letters were filtered to include communications between doctors, and only those letters that began with the token “Dear Dr” were included. The explicit bigram Dear Dr is a templated salutation that begins all clinical correspondence at BIDMC. The analysis was restricted to providers who had written a minimum of 10 letters before and after the OpenNotes debut date. The final list included 36 providers for initial and progress notes and 12 providers for letters (Table 1).

**TABLE 1.**
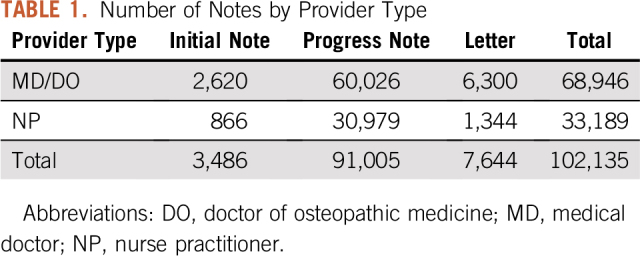
Number of Notes by Provider Type

### Definitions

Definitions of word, corpus, *n*-gram, distinctive/significant *n*-grams, and threshold are described in the Appendix.

### Cleaning and Preprocessing

The initial and progress notes were divided into two subcorpora: before November 25, 2013, and after. Preprocessing steps involve conversion of all text to lowercase, deletion of punctuation and new line tokens, splitting of the text on the basis of white space, and deletion of all words that are a single character in length (eg, a and I).^1^ We chose to avoid stemming for several reasons: Stems are hard to interpret in an actionable sense and although they are supposed to decrease word space, they may increase it by introducing hypothetical word stems that may potentially collide with actual words.

### *n*-Gram Algorithm

The algorithm begins with the selection of a provider and the analysis of the provider’s before and after corpora of initial and progress notes or letters by treating each corpus as a collection of 1-grams. All single words with frequencies less than Ω are eliminated (see Appendix). The corpus is then re-analyzed as a collection of 2-grams. Before analyzing the 2-grams, all 2-grams that do not consist of significant 1-grams are eliminated. The corpus is then analyzed as a collection of the remaining 2-grams, and all 2-grams with frequency less than Ω are eliminated. The analysis proceeds this way until the threshold requirement reduces the list of significant *n*-grams to 0 (described in the Appendix).

The data are analyzed using two complementary strategies. First, the proportions of use of each *n*-gram for each provider are analyzed in the before and after corpora. The proportion-of-use changes and their significance are determined using a simple statistical proportionality comparison test. Second, the providers are compared with one another using unsupervised cluster analysis to determine clusters of providers who use similar *n*-grams in their notes.

### Simple Proportional Use Analysis

All notes are re-analyzed for the presence or absence of each *n*-gram. A binary use matrix *U*, where *U*[*i*,*j*] = 0 or 1, is built. Any note *i* with at least one instance of the *n*-gram *j* is noted as present for that *n*-gram or *U*[*i*,*j*] = 1. Alternatively, if there are no instances of that *n*-gram, it is noted as absent or *U*[*i,j*] = 0. The proportion of use for each *n*-gram *j* is the sum of the column *j* divided by the number of rows.

Assessments are made whether a provider changes his or her use of an *n*-gram between the before and after OpenNotes subcorpora in a statistically significant way by testing the null hypothesis that no such change occurs. A two-sided 95% confidence proportion test (α/2 = .025) in the normal approximation is used. The SE is based on the pooled proportion. Only *n*-grams where the expected number of occurrences or nonoccurrences of a given *n*-gram on the basis of the pooled proportions before and after exceeds 10 are considered. This is guaranteed by the requirement of Ω = 0.10 and the number of notes before greater than 100 and number of notes after greater than 100.

### Clustering Algorithm

**FIG 1.**
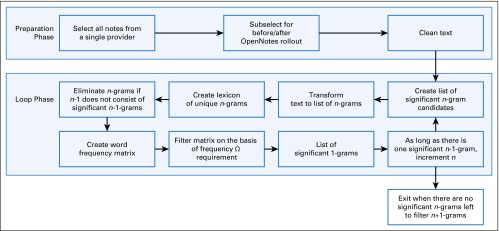
The protocol of cleaning the notes and building *n*-grams.

The providers’ list of *n*-grams represents sets that overlap when certain *n*-grams are used by two or more providers. These overlaps are used to perform unsupervised cluster analysis through a greedy aggregation algorithm^17^ (described in the Appendix). The lists of *n*-grams for all providers are compared, and the two that are the most similar on the basis of a similarity score are found. These two providers’ lists are merged, and the process is repeated until there is only one list left. The remaining list is the superset list of *n*-grams from which all provider lists can be sourced. Figure 1 summarizes the protocol.

## RESULTS

### Results for *n*-Gram Analysis

**FIG 2.**
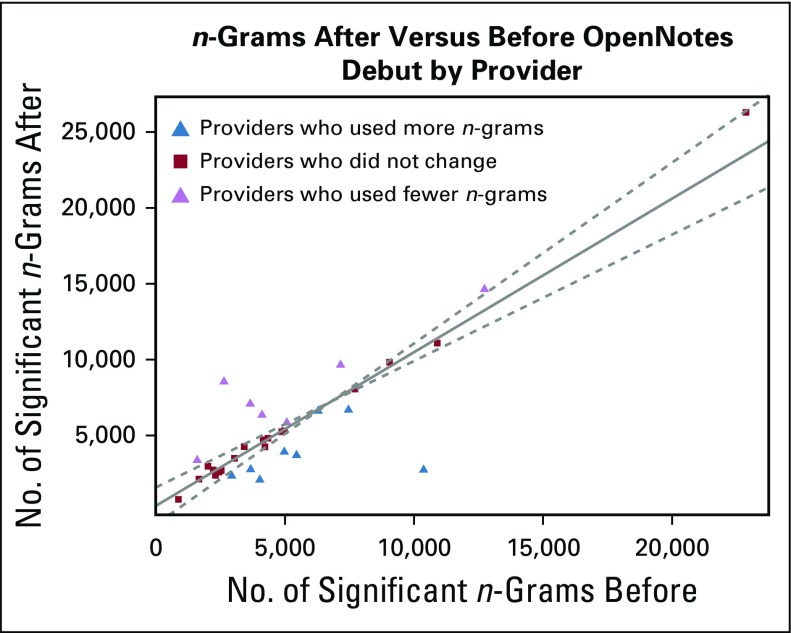
Number of primary and constituent *n*-grams used by providers in their initial and progress notes in the after corpus versus before corpus.

The cohort of 36 providers demonstrated several detectable shifts in *n*-gram use between the before and after corpora. A measure of note-writing behavior before versus after the OpenNotes debut is shown in Figure 2. On average, there was no significant change in the total number of *n*-grams used. The regression line is N_after = 1.01 × N_before + 80. The uncertainty in the slope is 0.08, and the uncertainty in the intercept is 560. The dashed lines indicate the 95% CI for the values around the regression line.

**FIG 3.**
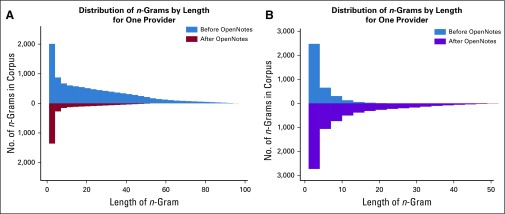
Distribution of lengths of primary and constituent *n*-grams before and after OpenNotes for two providers. (A) Provider A decreased creation of long *n*-grams after OpenNotes. (B) Provider B increased creation of long *n*-grams after OpenNotes.

Although the number of significant *n*-grams averaged over all providers did not change, approximately one half of the providers did have significant changes individually. Eight providers markedly decreased their use of significant *n*-grams, whereas nine increased their use. The observed effect was highly provider specific. For example, for one provider, the OpenNotes debut correlated with the decreased use of some long *n*-grams (Fig 3A). This provider was someone who significantly reduced reliance on certain long *n*-grams, specifically some templated text, such as the following:

96-gram: review_of_systems_negative_unless_marked_general_night_sweats_fever_chill_heent_oral_complaints_headache_visual_changes_or_sore_throat_cardiovascular_chest_pain_dizziness_palpitations_respiratory_cough_shortness_of_breath_or_wheezing_abdomen_abdominal_pain_nausea_vomiting_diarrhea_or_constipation_genitourinary_dysuria_or_change_in_urinary_pattern_ms_bone_pain_changes_in_muscle_strength_or_muscle_pain_skin_rashes_itching_endocrine_fatigue_frequent_urination_excessive_thirst_change_in_hair_texture_heme_lymph_easy_bruising_blood_clotting_or_bleeding_problems_increase_in_frequency_or_unusual_infections_neuro_psych_depression_si_hi_weakness_numbness_tingling_vertigo_physical_examination

As an example for another provider (Fig 3B), the debut of OpenNotes led to an increased dependence on longer *n*-grams. Most of this provider’s *n*-grams are associated with physical examination and prescription templates. After OpenNotes, this provider significantly increased use of longer *n*-grams, including:

32-gram: Full_affect_heent_clear_mucous_membranes_moist_sclera_anicteric_conjunctiva_pink_neck_soft_and_supple_chest_cta_no_wheezes_rales_or_rhonchi_heart_rrr_nl_s1_s2_abd_soft_nt_nd_bs

### Results for Clustering Analysis

**FIG 4.**
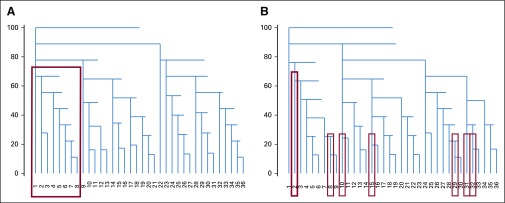
Dendrograms of the 36 included providers clustered by their use of similar *n*-grams. Red boxes show relatedness among the providers in the first cluster (late note signers). (A) Before the OpenNotes debut. (B) After the OpenNotes debut.

We observed some significant changes to the clustering structure of notes before and after the OpenNotes debut. The before dendrogram (Fig 4A) has three large clusters. The first cluster on the left consists of eight providers. This cluster is defined by 2,392 *n*-grams, which were used significantly by five of the eight providers. This indicates that these *n*-grams were 62% sensitive for that group. Ninety-five percent of those *n*-grams were also 100% specific to that group and, therefore, constituted distinctive *n*-grams to that cluster. All were constituent of the following 53-gram, which is the late signing attestation at BIDMC:

accurately_reflects_the_documentation_made_when_assessed_diagnosed_treated_and_or_communicated_about_the_above_named_patient_also_attest_that_this_information_is_true_accurate_and_complete_to_the_best_of_my_knowledge_and_understand_that_any_falsification_omission_or_concealment_of_material_fact_may_subject_me_to_administrative_civil_or_criminal_liability

The middle cluster of this dendrogram is not as easily interpreted. However, 944 *n*-grams distinguish it by being more than 60% sensitive and more than 80% specific. There were essentially no interpretatively distinctive *n*-grams in this group. In the third cluster, there were 1,214 *n*-grams that were at least 60% sensitive. Of these, 25 *n*-grams had at least 80% specificity. These *n*-grams seem to focus on prescriptions, for example: prochlorperazine_maleate_prochlorperazine_maleate_10_mg_tablet_tablet_by_mouth

After the debut of OpenNotes, the dendrogram and clusters were much more dispersed (Fig 4B). We observe that the eight providers in the first cluster from the first dendrogram have been scattered around the new set of clusters. We determined that this was due to the five late attesters having substantially reduced their late attestations after OpenNotes. Consequently, their relatedness dropped, and they were deemed closer to other providers on the basis of more subtle aspects of expression and usage. Of the five, one provider’s use of the late note attestation fell below the 10% threshold, and the corresponding *n*-grams were no longer significant. This result was confirmed with the proportional analysis of the 53-gram late note attestation. To summarize, unsupervised hierarchical clustering identified late note signers. Moreover, we can assert that late note signing was the largest driver of *n*-gram use variance in our study group.

### Proportional Analysis

The statistical test of comparison of proportions was used to determine whether the fractional use of certain *n*-grams increased or decreased significantly in the before and after corpora from provider to provider. In addition to the significant increase or decrease in proportion of use, the *n*-grams that rose above or fell below the threshold of significance (Ω) were also included.

**FIG 5.**
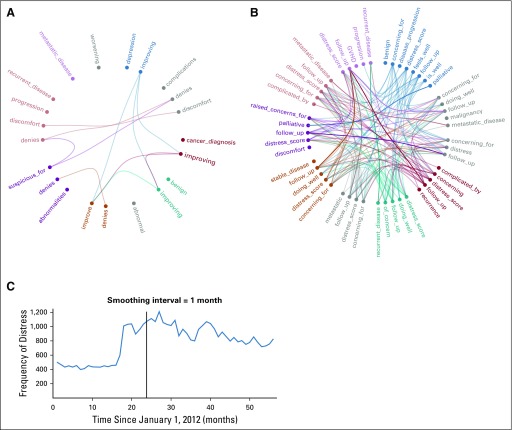
Relatedness of 10 providers by comparison of (A) increased and (B) decreased frequency of use of similar selected *n*-grams. The circle with segmented arcs in distinct colors indicates different providers. Each arc consists of several *n*-grams selected to indicate increase (or decrease) in use frequency. The lines that connect the *n*-grams emphasize the correlated increase (or decrease) in use frequency between providers for the specific word. (C) The occurrence of the word distress per 1,000 notes per month. Smoothing/aggregation interval = 1 month. The vertical line indicates the OpenNotes rollout date. GVHD, graft-versus-host disease.

Because not all *n*-grams are interpretable as tokens of valued medical communication, some value words were handpicked to display their increase or decrease in usage (Figs 5A and 5B). For example, the bigrams follow_up, distress_score, and concerning_for demonstrated a significant increase in frequency of use in most of the providers. The word distress_score was picked as a control because it was added as a policy to the vital signs sheet on April 11, 2013, and its use was encouraged coincident with the OpenNotes debut (Fig 5C). Thirty-four of 36 providers demonstrated significantly increased use of distress_score in the after corpus.

### Letter Analysis

Only five of 36 providers in the study wrote letters that started with the token Dear Dr and with sufficient abundance to guarantee more than 100 letters before and after the OpenNotes rollout. In one case, 403 *n*-grams appeared in both corpora, and only 88 changed proportion of use (*P* < .025). The use of the bigrams concerning_for and distress_score both increased significantly. For the others, 711 *n*-grams were used significantly in letters before and after the OpenNotes rollout. Of those, 113 changed use significantly, including follow_up and distress_score.

## DISCUSSION

The current study explored whether the introduction of OpenNotes changed documentation of patient encounters. We explored both the content and the related meta-data.

To analyze changes in providers’ notes content over time, we deconstructed medical notes on the basis of repeating sequential occurrences of words (*n*-grams) and modes of expression as seen in cluster/clique formation. Most providers used a combination of specific words, templates, and their own unique expressions when completing patient notes, and patterns in the unstructured content of these providers’ notes can be used to track documentation changes over time.

We have shown that although the number of distinct common *n*-grams did not change on average, nine providers dramatically increased their diversity of *n*-gram repertoire and eight contracted. Clustering analysis revealed that one driver for this change was the dramatic decrease in late note attestation by a subset of providers. Most observed changes were provider specific.

A previous effort to quantify redundancy in electronic medical records focused on 100 randomly selected patients admitted during a 6-month period at NewYork-Presbyterian Hospital in 2010. The study showed that more than 50% of notes (initial, progress, discharge, etc) borrowed extensively from previous notes from the same patient, which thereby indicates a cut-and-paste approach to note filling. Our approach differs significantly because we compared all notes from the same doctor with one another and not simply within one patient history. Therefore, we found features of doctors’ styles that are particular to the doctor rather than details that are particular to the patient.^18^

A major advantage of this novel method is the ability to detect the largest *n*-gram that is consistently used by providers in their notes. A second advantage of this method of *n*-gram construction is the independence from any prespecified lexicon. The need for predefined word lists, such as Unified Medical Language System, and the concomitant limitations was eliminated. A third advantage of this method is the use of aggregation clustering, which promotes the detection of sensitive and specific *n*-grams for clusters. Clusters are not merely mathematic or algorithmic associations. Rather, they are truly cohorts with shared communications modalities. A final advantage is the unsupervised nature of clustering itself. Future work will include an in-depth analysis of the similarities of providers grouped by cluster: Were they trained at similar institutions or during similar time periods? Do they share subspecialization in the field of hematology/oncology?

A limitation of this method assumes a static *n*-gram universe in each corpus. That is, the notes represent a sampling of a presumably time-independent distribution. Human writing, however, is not a static sampling, and as patients’ needs change from patient to patient and over time, the vocabulary shifts accordingly. The choice of a clustering metric is always arbitrary, and there may well be better metrics that give clusters with clearer delineations of sensitive and specific *n*-grams. Future work would focus on optimizing the metric for this purpose. However, the existing metric provides useful insights into shared *n*-gram usage. Similarly, the selection of primary *n*-grams rather than primary and constituent *n*-grams for analysis is an open question and depends strongly on the choice of clustering metric. We chose not to use stemming, which may have led to substantially different results that were due to errors in over- and understemming.^19,20^ Although vectorization has shown excellent results in many natural language processing (NLP) domains, written communication also relies heavily upon cadence, as introduced by punctuation. Thus, two similarly vectorized *n*-grams possibly would convey a different message to a patient. For example, “Mr Smith’s prognosis is dismal, given his age. We will proceed with aggressive chemotherapy” versus “Mr Smith’s prognosis is dismal. Given his age, we will proceed with aggressive chemotherapy.” Finally, *n*-grams are summary-level extractions of a document, which can be highly complex. Future work will focus on scaling this analysis to include prosody, tone, and reading-level metrics. Work on parsing the grammar of sentences has been done by the Stanford NLP project, among others. Recent work has sought to compare algorithms and assess for computational efficiencies. This work is an example of the state of the art of NLP, but it is beyond the scope of this article to implement.^21^

A limitation of the data source is that after exclusion, the universe of providers was relatively small and may have unmeasured biases that relate to the single institutional nature of the study. Of note, we analyzed notes at the provider level and did not focus on patient characteristics. Although the majority of patients seen in the hematology/oncology clinic have a diagnosis of cancer, the nature (curability) of their cancers may vary widely. We will explore the use of existing tools, such as DeepPhe,^22^ to delineate further the patient characteristics in future work.

In conclusion, we have performed an objective analysis of large corpora of hematology/oncology notes written before and after the OpenNotes rollout. Significant differences were seen in the content, which can be explained at least partially by the OpenNotes rollout.

## Appendix

### Definitions

#### Word, corpus, and *n*-gram.

A word is defined as a consecutive set of alphanumeric characters without punctuation or white space. Words with only one character in length, such as a or I, are ignored. A corpus includes all initial and progress notes or letters for a given provider. Provider notes were subdivided by the rollout date into two subcorpora: the before subcorpus and the after subcorpus.

An *n*-gram is a list of *n* consecutive words in a corpus. We avoid stems to record *n*-grams in higher resolution than stem analysis can provide. The most frequently used *n*-grams are commonplace terms, copy-and-paste or templated text, or provider-specific expressions. The least frequently used *n*-grams are single expressions that have little or no collective utility but often have substantial meaning in context. For example, if the text is “patient has very poor prognosis,” the 1-grams would be patient, has, very, poor, and prognosis, and the 2-grams would be patient_has, has_very, very_poor, and poor_prognosis. Any text of group of *n* words consists of (*n*) 1-grams, (*n* − 1) 2-grams, and so forth, up to (2) *n-*1*-*grams and (1) *n*-gram.

#### Significant *n*-grams and threshold.

A threshold of substantial meaning is defined by a threshold of substantial use (frequency of use in notes). The critical frequency of use Ω is the percentage of use above which the *n*-gram encodes information that is deemed common. Significant *n*-grams are a valid notion both for each provider’s own corpus and for the entire corpus. Here, we limit our focus on significance per provider. The union of significant *n*-grams per provider is a superset of *n*-grams that would have benefit in other studies.

The threshold was determined by using the normal approximation with a minimum of 100 notes before/after, a threshold of 10%. Therefore, Ω was chosen to be 10%; that is, a particular *n*-gram used in 10% of one provider’s notes is deemed significant.^20^

#### Primary and constituent *n*-grams.

Primary *n*-grams are significant *n*-grams that are not contained in a significant (*n*+1)-gram. Constituent *n*-grams are significant *n*-grams that are contained in a significant (*n* + 1)-gram.

#### Proportion.

The proportion of use of an *n*-gram is the fraction of notes in the corpus (before or after) that contains at least one instance of that *n*-gram.

#### Cluster.

A cluster is a subset of providers who mutually use large numbers of significant *n*-grams.

#### Distinctive *n*-grams.

Distinctive *n*-grams are sensitive and specific to a cluster of providers. The sensitivity of an *n*-gram is the fraction of providers in that cluster who use the given *n*-gram. The specificity of an *n*-gram is the fraction of providers not in that cluster who do not use the given *n*-gram.

#### Cluster algorithm.

The providers’ list of *n*-grams represents sets that overlap when certain *n*-grams are used by two or more providers. We use these overlaps to perform unsupervised cluster analysis through aggregation. We compare the lists of *n*-grams for all providers and find the two that are most similar on the basis of a similarity score. We merge these two provider lists and repeat the process until there is only one list left. The remaining list is the superset list of *n*-grams from which all providers lists can be sourced.

The analysis of each provider results in a list of *n*-grams. Each provider’s *n*-gram record contains the unique identifier of the provider, the *n*-grams themselves, and a score associated with each *n*-gram that counts the group-wise frequency of use (or popularity) of the *n*-gram. At the beginning of clustering, the popularity scores are initialized to unity for each *n*-gram in each provider’s list of *n*-grams.

We read the results of the *n*-grams analysis for each of the providers and store them as a list of lists. We then assess the similarity between each pair of the providers’ *n*-grams and determine a metric of similarity given by the following protocol.

For determining the similarity of lists, let *A* be a list of *n*-grams from provider A and *B* be a list of *n*-grams from provider B. Let *M* be the merged list of *n*-grams from providers A and B. *M* consists of the union of *A* and *B*, and it contains *N_M_* elements. The score of *n*-gram *i* in *M* is the sum of the scores of *n*-gram *i* in *A* and *B*. That is, if an *n*-gram occurs once in *A* and once in *B*, then its score is 2 in the merged list. If it appears in either *A* or *B*, then the score is 1.

The similarity of *A* and *B* is given by the ratio of two numbers. The numerator is the sum of the scores of the *n*-grams in *M* minus its number of *n*-grams *N_M_*. The maximal value of the numerator is 2*N_M_* − *N_M_* = *N_M_*. The minimal value of the numerator is 0 − *N_M_* = −*N_M_*. The denominator is the maximal value that the numerator may have. The maximum similarity score is 1, which occurs when the two lists are identical. The minimum score is −1 where the two lists have no intersection.

After selecting the most similar lists for merging, the lists *A* and *B* are removed from our analysis, and the list *M* is inserted in their place. The number of lists we analyze drops by one. Even though *M* represents the *n*-grams of two providers, we treat *M* as if it were a single provider in subsequent analysis. This means that if we want to compare provider C with *M*, we create the newly merged list *MC* with length *N_MC_*. Our metric of comparison will use the score values for *M* (1 or 2) and for *C*. If *C* is a basic list, its *n*-gram scores are 1. This adds a weight to the shared *n*-grams, making them more important for similarity than *n*-grams that are not shared. The maximal value of the numerator is now 3*N_MC_* − *N_MC_* = 2*N_MC_*, so the denominator becomes 2*N_MC_*, where *N_MC_* is the number of elements in the union of *M* and *C*. We continue to merge the most similar lists by inserting the merged list in their place until there is only one list left.

The resulting dendrogram is a binary tree. With *n* providers, there are *n*-1 merge events and therefore *n*-1 clusters. If the unsupervised clustering merges on the basis of similarity, then the resulting dendrogram will have subclusters of providers whose *n*-grams are similar. Failure to observe subgroups is an indication that either the metric of similarity is insufficient or that distinct subgroups simply do not exist.

#### Clusters and distinctive *n*-grams.

The value of unsupervised aggregation clusters is their ease of use in discovering sensitive and specific *n*-grams for the observed clusters and subclusters. Each node in the cluster tree represents a summary of all leaf nodes that lie beneath it. The sensitivity is the number of providers in a subcluster who use an *n*-gram divided by the number providers in that subcluster.

## References

[1] Gillum RF From papyrus to the electronic tablet: A brief history of the clinical medical record with lessons for the digital age Am J Med 126 853 857 2013 24054954 10.1016/j.amjmed.2013.03.024

[2] Doyle-Lindrud S The evolution of the electronic health record Clin J Oncol Nurs 19 153 154 2015 25840379 10.1188/15.CJON.153-154

[3] Gutheil TG Fundamentals of medical record documentation Psychiatry (Edgmont Pa) 1 26 28 2004 PMC3010959 21191523

[4] Thomas J Medical records and issues in negligence Indian J Urol 25 384 388 2009 19881136 10.4103/0970-1591.56208 PMC2779965

[5] West SL Johnson W Visscher W et al The challenges of linking health insurer claims with electronic medical records Health Informatics J 20 22 34 2014 24550563 10.1177/1460458213476506

[6] Walker J Delbanco T Interval examination: Moving toward open notes J Gen Intern Med 28 965 969 2013 23620188 10.1007/s11606-013-2407-3 PMC3682039

[7] Ross SE Lin CT The effects of promoting patient access to medical records: A review J Am Med Inform Assoc 10 129 138 2003 12595402 10.1197/jamia.M1147 PMC150366

[8] Annas GJ: A.H.A. Bill of Rights. Trial 9:59-61, 1973 11662033

[9] Department of Health and Human Services: Standards for privacy of individually identifiable health information. Billing Code 4150-04 M (45 CFR Parts 160 and 164). Fed Regist 65:82461-82829, 2000

[10] Lye CT, Forman HP, Gao R, et al: Assessment of US Hospital Compliance With Regulations for Patients’ Requests for Medical Records, 2018. https://jamanetwork.com/journals/jamanetworkopen/fullarticle/2705850 10.1001/jamanetworkopen.2018.3014 PMC6324595 30646219

[11] Masys D Baker D Butros A et al Giving patients access to their medical records via the Internet: The PCASSO experience J Am Med Inform Assoc 9 181 191 2002 11861633 10.1197/jamia.M1005 PMC344575

[12] Ross SE Moore LA Earnest MA et al Providing a Web-based online medical record with electronic communication capabilities to patients with congestive heart failure: Randomized trial J Med Internet Res 6 e12 2004 15249261 10.2196/jmir.6.2.e12 PMC1550594

[13] Delbanco T Walker J Darer JD et al Open notes: Doctors and patients signing on Ann Intern Med 153 121 125 2010 20643992 10.7326/0003-4819-153-2-201007200-00008

[14] Delbanco T Walker J Bell SK et al Inviting patients to read their doctors’ notes: A quasi-experimental study and a look ahead Ann Intern Med 157 461 470 2012 23027317 10.7326/0003-4819-157-7-201210020-00002 PMC3908866

[15] OpenNotes: See Who’s Already Sharing Notes! https://www.opennotes.org/join/map

[16] Jain SK, Rahimian M, Zerillo J, et al: Using network graphs to visualize changing documentation styles in an oncology practice before and after OpenNotes implementation, in IEEE Workshop on Visual Analytics in Healthcare (VAHC), Phoenix, AZ, 2017, pp 62-68

[17] Conway GC Smole SC Sarracino DA et al Phyloproteomics: Species identification of Enterobacteriaceae using matrix-assisted laser desorption/ionization time-of-flight mass spectrometry J Mol Microbiol Biotechnol 3 103 112 2001 11200222

[18] Wrenn JO, Stein DM, Bakken S, et al: Quantifying clinical narrative redundancy in an electronic health record. J Am Med Inform Assoc 17:49-53, 2010 10.1197/jamia.M3390 PMC2995640 20064801

[19] Jivani A: A comparative study of stemming algorithms. Int J Comp Tech Appl 2:1930-1938, 2011

[20] Rajput BS, Khare N: A survey of stemming algorithms for information retrieval. IOSR J Comput Engineer 17:76-80, 2015

[21] Kong L, Smith NA: An empirical comparison of parsing methods for Stanford dependencies, 2014. https://arxiv.org/pdf/1404.4314.pdf

[22] Savova GK Tseytlin E Finan S et al DeepPhe: A natural language processing system for extracting cancer phenotypes from clinical records Cancer Res 77 e115 e118 2017 29092954 10.1158/0008-5472.CAN-17-0615 PMC5690492

